# Functions of Macrophages in the Maintenance of Intestinal Homeostasis

**DOI:** 10.1155/2019/1512969

**Published:** 2019-03-18

**Authors:** Shuai Wang, Qianhong Ye, Xiangfang Zeng, Shiyan Qiao

**Affiliations:** ^1^Department of Animal Nutrition and Feed Science, College of Animal Science and Technology, Huazhong Agricultural University, Wuhan, Hubei 430070, China; ^2^State Key Laboratory of Animal Nutrition, Beijing Key Laboratory of Biofeed Additives, Ministry of Agriculture Feed Industry Centre, China Agricultural University, Beijing 100193, China

## Abstract

Intestinal macrophages constitute the largest pool of macrophages in the body and have emerged as crucial sentinels for pathogen recognition and elimination. The source and development of intestinal macrophages, as well as their distinct properties have been well documented. Intestinal macrophages exert their functions in the maintenance of intestinal homeostasis by shaping host-microbiota symbiosis, managing gut inflammation, crosstalking with T cells, and facilitating wound repair. Recently, nutritional regulation of intestinal macrophages has attracted substantial attention and is becoming a promising approach to disease prevention and control. Understanding the mechanisms employed by intestinal macrophages in mediating intestinal immune homeostasis and inflammation, as well as the mode of action of dietary nutrients in the modulating functions of intestinal macrophages, represents an opportunity to prevent and control inflammatory bowel diseases.

## 1. Introduction

The gastrointestinal tract mucosa is continually exposed to a high load of antigens, ranging from dietary proteins and commensal microbiota to clinically important pathogens, viruses, and toxins. A single layer of intestinal epithelial cells form a barrier between the lamina propria and the luminal contents of the intestine. Intestinal macrophages that reside in the subepithelial lamina propria (LP) represent the most abundant mononuclear phagocytes in the body and have emerged as crucial sentinels for the maintenance of intestinal homeostasis [1]. As the first phagocytic cells of the innate immune system, intestinal macrophages engulf and clear pathogens, cellular debris, and bacterial products, constantly maintaining a balance between immunity against foreign pathogens and tolerance to commensals [2]. Nonetheless, the cellular and molecular mechanisms by which this critical balance is achieved remain relatively unknown. Due to the crucial role of macrophages in the initiation and development of intestinal immunity, therapeutically manipulating macrophages are becoming an attracting way for disease prevention and treatment. In this review, we focus our attention on intestinal macrophages, describing the recent insights into the role of intestinal macrophages in maintaining gut homeostasis and managing gut inflammation. Finally, we will discuss the nutritional modulation of intestinal macrophage function and the potential of nutritional strategies aimed at manipulating intestinal macrophages to ameliorate inflammatory bowel disorders.

## 2. Intestinal Macrophages

Intestinal macrophages, which constitute the largest pool of macrophages in the body, are the most abundant mononuclear phagocytes in the LP. Macrophages in the intestine are identified by the expression of F4/80 and CD64 markers, as well as the integrin CD11b [3, 4]. Mature intestinal macrophages also express high levels of the chemokine receptor CX3CR1 [5]. However, with the deepening research on the intestinal mucosal immune system, these characteristic markers have not been able to distinguish intestinal macrophages from other cells. For instance, dendritic cells share many phenotypic characteristics with macrophages, such as MHCII and CD11b [6, 7]. Thus, additional markers need to be discovered to distinguish intestinal macrophages from other cells.

## 3. Source and Development of Intestinal Macrophages

Intestinal macrophages, which are thought to play a pivotal role in orchestrating intestinal mucosal immune responses, have received relatively little research attention compared with other tissue macrophages. Macrophages are present in virtually the entire body. In contrast to macrophages from many other tissues, those in the LP of the intestine are continuously replenished from recruited Ly6C^+^ blood monocytes under steady state or in response to inflammation [8]. These peripheral-blood monocytes develop from hematopoietic stem cells in the bone marrow. During monocyte development, hematopoietic stem cells divide and differentiate in to monoblasts, then promonocytes, and finally monocytes in the presence of macrophage colony-stimulating factor [9]. The CCL2-CCR2 axis plays a critical role in the migration of Ly6C^hi^ monocytes from the bone marrow to the peripheral blood [10, 11]. Bone-marrow monocytes have been classified into two principal subsets with distinct migratory properties in mice [12]. In steady state condition, Ly6C^hi^ monocytes enter the gut mucosa and differentiate into mature CX3CR1^hi^F4/80^+^ macrophages via a CX3CR1^int^ transitional stage. These CX3CR1^hi^ macrophages produce PGE2 and help maintain integrity of the gut epithelial layer [13]. Additionally, CX3CR1^hi^ macrophages also secrete interleukin-10 (IL-10), an anti-inflammatory cytokine that maintains mucosal homeostasis [14, 15]. Likewise, lamina propria macrophages drive differentiation of regulatory T (T_reg_) cells in the intestinal mucosa through production of IL-10 [16]. Signaling mediated by the IL-10 receptor plays a pivotal role in the hyporesponsiveness of murine or human intestinal macrophages. Macrophage-derived IL-10 also maintains survival and expansion of inducible FoxP3^+^ T_reg_ cells in the LP, which are crucial for tolerance of orally ingested antigens in mice [17]. Impaired production of IL-10 would result in macrophage hyperactivity and inflammatory bowel disease in mice and humans [18, 19]. The IL-10–IL-10R axis, especially IL-10 receptor, is indispensable for gut homeostasis. Macrophages unable to sense IL-10, due to loss of IL-10 receptor, play a central role in the development of severe spontaneous colitis [20]. When intestinal homeostasis is disturbed by infection or inflammation, the normal pattern of monocyte differentiation is disrupted. Ly6C^hi^ monocytes and their CX3CR1^int^ derivatives are recruited to the intestinal mucosa in large numbers during incidents of acute colitis [6]. The CX3CR1^int^ macrophages produce large amounts of TNF-*α*, IL-6, IL-12, and IL-23, as well as iNOS, rendering them responsive to TLR stimulation to become proinflammatory effector cells [5, 21, 22]. In addition, Ly6C^hi^ monocytes may recruit other innate effector cells via production of chemokines. For example, Waddell et al. (2011) found that Ly6C^hi^ monocytes orchestrated the recruitment of eosinophils through secretion of CCL11 (eotaxin) in a mouse model of dextran sodium sulfate- (DSS-) induced colitis. Importantly, these elicited Ly6C^hi^ monocytes are able to directly control the pathogenic effects of neutrophils and, in particular, the production of TNF-*α* and ROS by neutrophils in a PGE2-dependent manner [13].

## 4. The Distinct Properties of Intestinal Macrophages

The epithelial surface of the gastrointestinal tract is exposed to a great mass of bacteria as well as a large number and diversity of dietary antigens. The primary role of intestinal macrophages is to act as innate effector cells in the intestinal LP. To cope with this large antigenic load that may potentially cross the intestinal LP, macrophages in the intestine form some functional adaptations to preserve local tissue homeostasis [23]. Unlike their progenitor cells and blood monocytes, human intestinal macrophages show greatly diminished expression of costimulatory molecules, such as CD40, CD80, and CD 86 [24]. In addition, human resident intestinal macrophages exhibit greater phagocytic activity without initiating an inflammatory response due to their low, or even absent, expression of innate response receptors, including receptors for LPS (CD14), Fc*α* (CD89), Fc*γ* (CD64, CD32, and CD16), CR3 (CD11b/CD18), and CR4 (CD11c/CD18) [25]. This hyporesponsiveness enables intestinal macrophages to act as efficient scavengers without inducing inflammation that would normally occur and impair intestinal homeostasis when macrophages encounter pathogens. Finally, human intestinal macrophages also lack the triggering receptor expressed on myeloid cells-1 (TREM-1) [26]. TREM-1 is a cell surface molecule expressed on peripheral blood neutrophils and monocytes/macrophages. This cell surface molecule is an efficient amplifier of inflammation because TREM-1-mediated activation causes enhanced expression of proinflammatory mediators (e.g., TNF, IL-1*β*, and IL-6) or an upregulation of several cell surface molecules indicating oxidative burst (e.g., CD40, CD86, and CD 32) [27]. The absence of TREM-1 expression on human intestinal macrophages probably contributes to the low level of inflammation observed under physiological conditions, which can be regarded as a further adaptation of intestinal macrophages to the specific environment of the intestinal LP.

## 5. Functions of Intestinal Macrophages

### 5.1. Shaping Host-Microbiota Symbiosis

**Figure 1:**
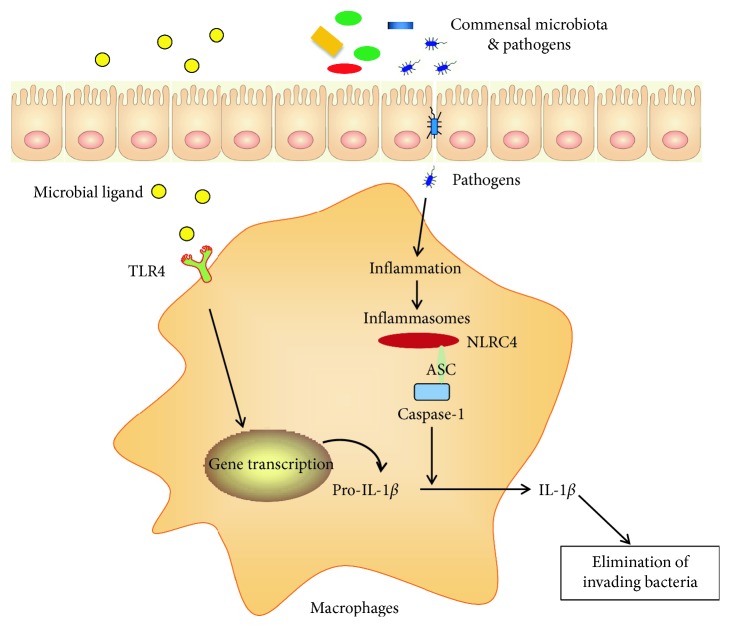
The role of intestinal macrophages in shaping host-microbiota symbiosis. The gastrointestinal tract is colonized by a dynamic community of microorganisms. Intestinal macrophages are anergic to microbial ligand from commensals and consistently produce a precursor to interleukin 1*β* (pro-IL-1*β*). Nod-like receptor NLRC4 and the adaptor ASC are crucial components of inflammasome by transmitting pathogenic danger signals to caspase-1 activation. Active caspase-1 is essential for the cleavage of pro-IL-1*β* into its mature and biologically active form. Mature IL-1*β* is critical in the elimination of invading bacteria in the intestine.

Given the trillions of microorganisms that live in the intestine, the intestinal immune system must continually sustain a balance between immunity to pathogens and tolerance of commensals to prevent needless immune responses against inoffensive bacteria. A question arises about how the immune system discriminates between pathogenic and commensal bacteria. One explanation is that the immune system can discriminate between commensals and pathogens through recognition of symbiotic microbial molecules. *Bacteroides fragilis* is a prominent gut commensal. The symbiosis factor, polysaccharide A (PSA) of *B. fragilis*, is essential for *B. fragilis* to suppress T-helper 17 (Th17) responses during homeostatic colonization [28]. In addition, resident macrophages are hyporesponsive to Toll-like receptor (TLR) stimulation but constantly produce pro-IL-1*β*, whereas pathogens but not commensals could elicit mature IL-1*β* through the NLRC4 inflammasome. Inflammasomes are molecular platforms inducing the activation of caspase-1, which lead to the secretion of mature and biologically active IL-1*β* [29] (Figure 1). Additionally, intestinal macrophages can also help maintain intestinal homeostasis by inducing production of anti-inflammatory cytokines, as well as engulfing and degrading commensals [25].

### 5.2. Managing Gut Inflammation

An increasing body of evidence suggests that macrophages located in the intestinal mucosa have an important role in maintaining the tolerance of commensals while staying responsive to pathogens [2]. However, disorders in enteric bacterial recognition by intestinal macrophages can result in chronic intestinal inflammation, such as inflammatory bowel diseases (IBDs) [30]. Proinflammatory macrophages (CX3CR1^int^ cells) isolated from an inflamed intestine produce large amounts of IL-1*β*, IL-6, TNF-*α*, IL-23, and NO [13, 31–33]. Besides contributing to tissue damage, these factors mediate the bactericidal function of macrophages. TNF-*α* has many functions such as activation and chemotaxis of neutrophils to kill microbes [34]. NO, synthesized by iNOS, is a short-lived gas that possesses beneficial roles in antibacterial activity of macrophages against pathogens [35]. Heme-oxygenase-1 (HMOX-1) is an antioxidant and anti-inflammatory enzyme produced by CX3CR1^+^ macrophages. Previous studies reported that HMOX-1 also helps to control inflammation in the intestine via enhancing phagocytic activity of macrophages [36]. It is well recognized that IL-23 is essential for host defense during the early phase of infection. For example, during the early phase of *Citrobacter rodentium* infection, invasion of the pathogen leads to secretion of IL-23 [37]. IL-23 can stimulate IL-22 production under several infectious conditions [38], and IL-22 seems to be indispensable in protecting the integrity of the intestinal epithelial layer. IL-22 also plays a key role in preventing the spread of pathogens by inducing antimicrobial peptides and chemokines that recruit immune cells to the site of infection [39]. Therefore, proinflammatory intestinal macrophages are essential for protection against pathogenic bacterial infections such as salmonellosis and colibacillosis [25, 40].

### 5.3. Crosstalk with T Cells

Macrophages can also maintain immunological homeostasis via induction or expansion of regulatory T cells in the intestine [41, 42]. FoxP3^+^ T_reg_ cells play a critical role in intestinal homeostasis. Mice deprived of T_reg_ cells are more susceptible to colitis [43]. In the LP, CD11^b+^F4/80^+^CD11^c−^ macrophages induce differentiation of FoxP3^+^ T_reg_ cells via a mechanism dependent on retinoic acid, IL-10, and transforming growth factor-*β* (TGF-*β*) [16]. In parallel, the number of FoxP3^+^ T_reg_ cells in the intestine is correlated with macrophage numbers [44]. Moreover, these FoxP3^+^ T_reg_ cells have also been reported to have the ability to inhibit inflammatory activity of Th1 and Th17 cells in inflamed intestines [45]. Collectively, these studies emphasize the function of macrophages as a bridge between innate and adaptive immunity against infections in the intestine.

### 5.4. Wound Repair

**Figure 2:**
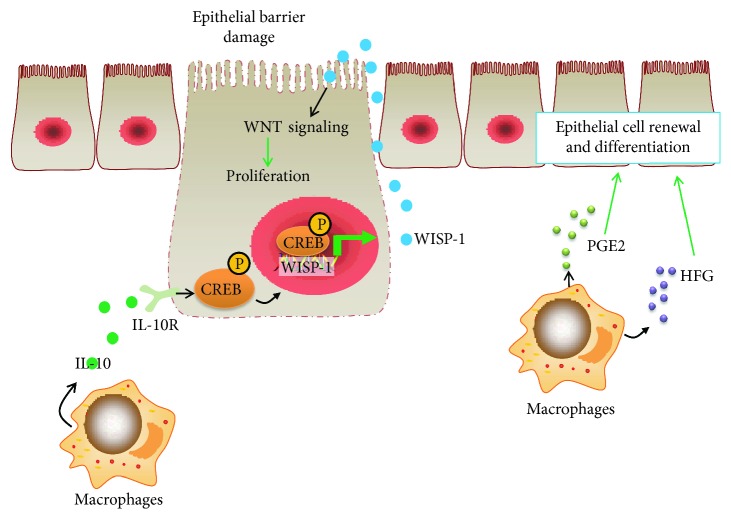
Macrophages contribute to the coordination of wound healing. Macrophages recruited to the sites of intestinal injury produce IL-10, resulting in the activation of cAMP response element-binding protein (CREB) signaling. This signaling enhances secretion of WNT-1-inducible signaling protein 1 (WISP-1) that in turn promotes WNT signaling, epithelial cell proliferation, and wound healing in the intestine. Additionally, intestinal macrophages also secrete prostaglandin E2 (PGE2) and hepatocyte growth factor (HGF), which stimulate renewal and differentiation of the intestinal epithelium.

Epithelial damage concerned with the impairment of the intestinal mucosal layer occurs following mechanical injury and is a characteristic of inflammatory bowel disease. Repair of the mucosal layer is crucial for alleviating gut inflammation and regaining intestinal homeostasis. Macrophages contribute to the coordination of tissue repair [46] (Figure 2). Macrophages are a major source of IL-10 for healing intestinal mucosa. IL-10 activates the cAMP response element-binding protein (CREB) signaling. This signaling promotes secretion of WNT1-inducible signaling protein 1 (WISP-1) that in turn enhances *β*-catenin/TCF signaling, epithelial cell proliferation, and repair in the intestine [46]. In addition, Cosín-Roger et al. found that the STAT6-dependent macrophage phenotype accelerated wound healing in the intestinal mucosa of mice treated with 2,4,6-trinitrobenzenesulfonic acid (TNBS) through eliciting the Wnt signaling pathway [47]. Moreover, macrophages have been reported to secrete several mediators including PGE2 and hepatocyte growth factor that can promote renewal and differentiations of intestinal epithelial cells [48, 49]. However, the role of macrophages is a double-edged sword for health. Intestinal macrophages have been reported to be crucial determinants of gut carcinogenesis [50]. Tumor-associated macrophages efficiently trigger angiogenesis that provides nutrition and oxygenation to tumor cells [50]. A better understanding of the modulation of macrophage phenotypes and their tumor-promoting functions would contribute to a promising design of tumor-associated macrophage-centered therapeutic interventions.

## 6. Influence of Nutrition on Intestinal Macrophage Function

An important role for enteral nutrients in modulation of intestinal macrophages is emerging. Many diet-derived luminal metabolites that are processed by gut microbiota, such as short-chain fatty acids (SCFAs), vitamins, and bile acids, have been demonstrated to regulate immune cell functions in the intestine. In addition, certain nutrients derived from the diet, without processing by microbiota, also possess immunomodulatory functions [51, 52]. Not surprisingly, the effects of dietary nutrients on the regulation of intestinal macrophages have attracted substantial attention in recent years.

### 6.1. Fatty Acids

**Figure 3:**
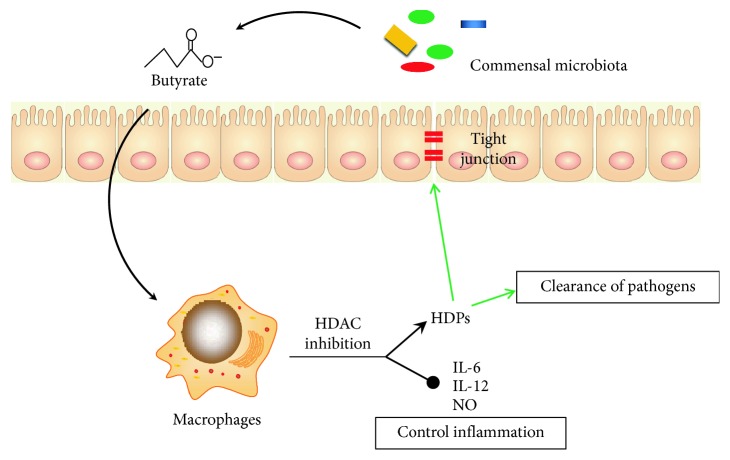
Mechanism of butyrate in modulating intestinal macrophage function. The microbial metabolite butyrate causes intestinal macrophages to reduce secretion of proinflammatory mediators such as IL-6, IL-12, and nitric oxide (NO) via inhibition of histone deacetylase (HDAC). This effect drives the intestinal immune system to be tolerant of commensals. Butyrate is also a strong inducer of host defense peptides (HDPs) that have pleiotropic functions in the maintenance of intestinal homeostasis, such as upregulation of tight junction protein expression in the intestinal epithelium, and clearance of pathogenic bacteria.

Short-chain fatty acids (SCFAs) including acetate, propionate, and butyrate are metabolites of gut bacterial fermentation of dietary fiber that are not digested by host in the small intestine [53]. Increasing evidence suggests that SCFAs have a potential to modulate the immune response in the intestine. Administration of SCFA can alleviate intestinal inflammation and lesions in patients with colitis or in murine colitis models [54, 55]. These immunomodulatory effects of SCFA are probably due to their anti-inflammatory properties [56–58]. Recent work has demonstrated that butyrate can modulate intestinal macrophage function, thereby contributing to homeostasis in the intestines [2] (Figure 3). Treatment of macrophages with butyrate results in downregulation of LPS-induced proinflammatory mediators, such as IL-6, IL-12, and nitric oxide. These effects are attributed to inhibition of histone deacetylases by butyrate [2].

### 6.2. Functional Amino Acids

A deficiency of dietary amino acids is known to cause malnutrition and then impair the intestinal immune system, increasing susceptibility of the host to infectious disease. Accumulating evidence indicates that dietary amino acids have the capability of regulating intestinal macrophage functions [52, 59]. For instance, deprivation of enteral nutrients related to total parenteral nutrition results in a decrease in the number of IL-10-producing macrophages in the small intestine of mice. Whereas dietary amino acids are able to directly regulate replenishment of intestinal macrophages and their IL-10 secretion [52]. However, further studies are needed to elucidate the mechanism by which dietary amino acids modulate macrophage function. It was found that dietary histidine prevented the development of colitis in an IL-10-deficient murine model. The protective effects of histidine were due to its suppression of NF-*κ*B activation in macrophages, thereby inhibiting the production of proinflammatory cytokines such as TNF-*α* and IL-6 [59]. Furthermore, previous studies have demonstrated that specific amino acids, such as arginine and glutamine, are required for the phagocytic activity of macrophages [60, 61]. Oral administration of tryptophan has been shown to promote phagocytosis by macrophages, which might contribute to increased resistance to pathogenic infections in rats [62]. New knowledge about the role of amino acids in regulation of intestinal macrophage function is important for the development of effective strategies to prevent immunodeficient diseases.

### 6.3. Vitamins

Vitamin A and its derivative, retinoic acid (RA), modulate a broad spectrum of immune functions. Retinoic acid, the active metabolite of vitamin A, is produced by many subsets of intestinal antigen-presenting cells (APCs) including macrophages and dendritic cells. It has been recognized for decades that vitamin A insufficiency is related to increased susceptibility to various types of infections and impairment of both the innate and adaptive immune systems [63, 64]. Emerging evidence demonstrates that RA has an indispensable role in modulating the functions of APCs in the intestine [65, 66]. Wang et al. reported that RA suppressed IL-12 production while increasing IL-10 production in macrophages [67]. However, vitamin A deficiency was found to exacerbate inflammation in a rat model of colitis [68]. In addition, vitamin A deficiency decreased phagocytic activity and bactericidal capacity of macrophages [65]. Nevertheless, oral administration of RA can inhibit in vivo growth of *Mycobacterium tuberculosis* via downregulating tryptophan-aspartate-containing coat protein (TACO) gene transcription [69]. A previous study demonstrated that downregulation of TACO gene transcription can restrict entry/survival of *M. tuberculosis* in macrophages [70].

Vitamin D is also a strong modulator for macrophage functions. Zhang et al. found that vitamin D suppressed the production of proinflammatory cytokines in macrophages via targeting MAPK phosphatase-1 [71]. In addition, vitamin D(3)-1,25-dihydroxyvitamin D(3) directly stimulates the host defense peptide cathelicidin expression through the vitamin D receptor, which is required for the antimicrobial activity against *M. tuberculosis* in macrophages [72, 73]. Host defense peptides (HDPs) constitute an important component of the innate immune system and provide immediately effective and nonspecific defenses against infections [74]. Oral supplementation of compounds that induce HDP synthesis has recently become a novel and promising strategy to prevent and control infections in both humans and animals [75, 76]. Myeloid cells, especially macrophages and neutrophils, are major sources of most HDPs. Therefore, the induction of HDPs represents another important mechanism in enhancing macrophage function by vitamin D.

## 7. Conclusion

Macrophages are indispensable modulators of the innate immune system because they maintain a delicate balance between immunity against pathogenic bacteria and tolerance of commensals in the intestine. Nutritional modulation of intestinal macrophages is becoming a promising approach to disease prevention and has attracted considerable attention. A better understanding of mechanisms employed by intestinal macrophages in maintaining intestinal homeostasis and the action of enteral nutrients in the regulation of intestinal macrophages will facilitate the development of nutritional strategies in gut health improvement as well as prevention and control of inflammatory bowel disorders.
